# Gastric Cancer With Brain Metastasis: A Case Report

**DOI:** 10.7759/cureus.50040

**Published:** 2023-12-06

**Authors:** Ethan Kosco, Noah King, Andrew Waack, Alastair Hoyt, Jason Schroeder

**Affiliations:** 1 Medicine, The University of Toledo College of Medicine and Life Sciences, Toledo, USA; 2 Neurological Surgery, The University of Toledo Medical Center, Toledo, USA

**Keywords:** genetic, lynch syndrome, mri, radiation, metastasis, gastric cancer

## Abstract

Although gastric cancer is one of the most common types of cancer worldwide, it rarely involves metastasis to the brain. Brain metastases can present with non-specific neurological symptoms such as focal neurological deficits, personality changes, or ataxia. Unfortunately, once brain metastasis is confirmed using imaging, the average life span is approximately two to four months. However, surgical and nonsurgical interventions have been able to improve quality and extend life to up to a year in patients living with gastric cancer that has metastasized to the brain. We report the diagnosis and surgical management of a 73-year-old female who presented with brain metastasis from gastric cancer. After a combination of radiation therapy, surgical management, and pharmacological intervention, the metastasis was successfully removed from the brain, as indicated by a negative CT and MRI on a four-year follow-up.

## Introduction

Gastric cancer is the fifth most commonly diagnosed cancer worldwide, with over one million new cases diagnosed each year [1]. This form of cancer commonly metastasizes to the peritoneum, lymph nodes, and liver [2]. However, an extremely rare manifestation of late-stage gastric cancer is metastasis to the brain; less than 1% of gastric cancers spread to the brain, and the associated median survival at this stage of the disease course is approximately two to four months [3-6]. Depending on the region of the brain affected by the malignant cells, gastric cancer-associated brain metastasis can present with personality changes, cachexia, ataxia, or focal neurological deficits [3,7].

When used as an isolated therapy, modern treatment options such as surgical resection, stereotactic radiosurgery (SRS), whole-brain radiotherapy (WBRT), and chemotherapy have primarily improved symptoms rather than removed cancer [8,9]. Nevertheless, aggressive treatment with a combination of these therapies offers a survival advantage [9]. Therefore, gastric cancer with brain metastasis has been under-investigated. Additional research is warranted to determine the most effective combination of treatments that can be used to increase a patient’s quality of life and potential survival advantage. In this study, we report the case of a 73-year-old female who developed brain metastasis from gastric cancer.

## Case presentation

A 73-year-old woman with a past medical history of diabetes mellitus, lumbar radiculopathy, and arthrodesis presented to the emergency room following one right upper extremity clonic seizure with staring and progressive aphasia for the past month. She had a history of unresectable metastatic gastric adenocarcinoma T4N2M0, IV first diagnosed five months ago in December 2019. An extensive family history of cancer warranted genetic testing, which revealed an MSH6 pathogenic mutation (Lynch syndrome). At the time of the presentation, she had received 10 fractions of stereotactic body radiation therapy (SBRT) for the primary unresectable gastric cancer and four cycles of capecitabine and decadron.

**Figure 1:**
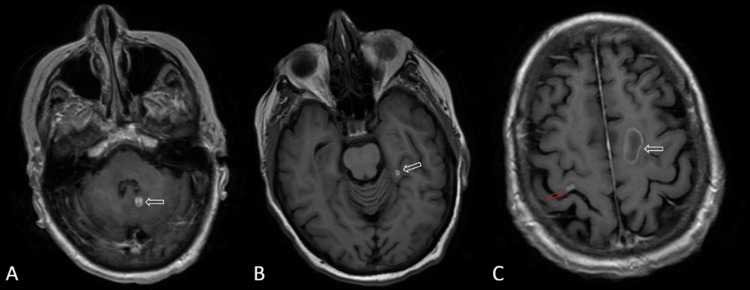
Post-contrast axial T1 imaging. MRI images of gastric metastasis at initial discovery including the medial aspect of the left cerebellar hemisphere (A), left medial temporal lobe (B), right precentral gyrus (C, red arrow), and left frontal lobe (C, white arrow).

A brain MRI revealed four diffusion-restricting, variably enhancing lesions with internal foci of susceptibility/hemorrhage and surrounding edema throughout the cerebral and cerebellar hemispheres (Figure 1). The largest lesion was in the left frontal lobe and measured 2.0 x 2.4 cm. Three smaller enhancing lesions measured at just <1 cm were located at the right precentral gyrus, left medial temporal lobe, and the medial aspect of the left cerebellar hemisphere. The discovery of brain metastasis prompted the oncologist to prescribe three fractions of SRS targeted on the largest lesion in the left frontal lobe and one fraction to each of the smaller lesions. At this point, the patient’s care was a joint effort between oncology and neurosurgery. She was switched to pembrolizumab monotherapy following completion of SRS in April 2020 considering positive Lynch syndrome. Post-radiation expressive aphasia, right-sided weakness, and recurring seizures were managed with levetiracetam.

**Figure 2:**
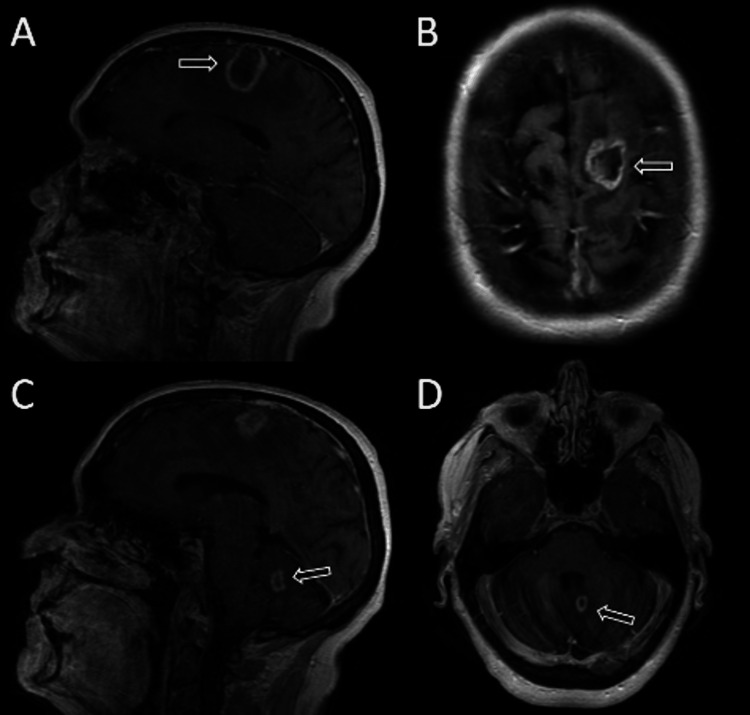
Post-contrast T1 imaging (A/C, sagittal; B/D, axial). MRI images of brain lesions at preoperative evaluation, one year after stereotactic radiosurgery, including left frontal lobe (A, B) and medial left cerebellar hemisphere lesion (C, D). Imaging findings were consistent with radiation necrosis.

**Figure 3:**
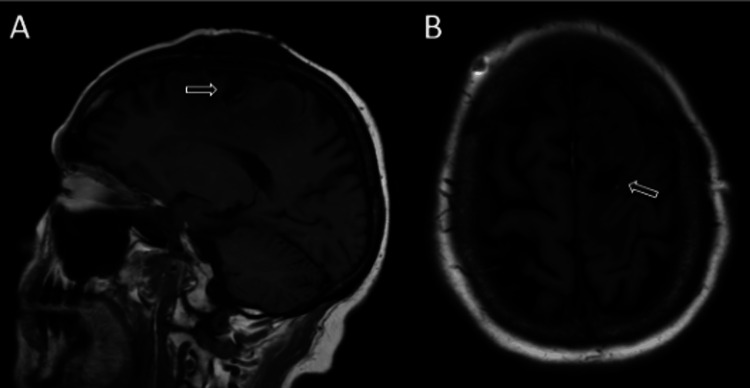
Sagittal T1 fluid-attenuated inversion recovery imaging (A, sagittal; B, axial). Postoperative MRI imaging demonstrating resection of left frontal lobe radiation necrosis.

One year later, the patient experienced increasing symptoms of headache, seizure, expressive aphasia, and right-sided weakness. Subsequent MRI revealed a slightly enlarged 1.9 x 2.8 cm left frontal lesion with progressively worsening cerebral edema and a singular 0.9 x 1.1 cm posterior fossa lesion (Figures 2, 3). The patient elected for left-sided craniotomy. Pathology report of the resected mass revealed radiation necrosis. The frequency of seizures vastly decreased but severe headaches remained following the procedure. The patient’s last dose of pembrolizumab was in January 2022, discontinued due to G3-4 fatigue, regular surveillance imaging was continued. Her most recent CT and MRI in July 2023 showed no evidence of disease recurrence or progression.

## Discussion

We present the case of a 73-year-old woman whose combination of pharmacologic and surgical management of brain metastasis from gastric cancer led to complete resolution of metastasis and neurological impairment at the four-year follow-up. Over one million cases of gastric cancer are diagnosed globally each year [1]. The condition is over twice as common in males compared to females and comprises 8.3% of all cancer deaths [1]. Despite the male predilection, mortality rates significantly rise around age 75 in both sexes [10]. Gastric cancer initially spreads to surrounding anatomical structures, such as the liver, peritoneum, and lymph nodes. Brain metastasis is a late manifestation of gastric cancer, as described by less than 1% of gastric cancers ultimately reaching the brain and the associated two to four-month survival rate at this point [3-5]. A retrospective study reported that 11 out of 2,322 (0.47%) patients diagnosed with gastric cancer between 1980 and 1998 had brain metastasis [11].

Gastroesophageal reflux disease, *Helicobacter pylori*, low socioeconomic status, genetics, and dietary factors are risk factors for gastric cancer that typically arise in the form of adenocarcinoma [1]. Of those risk factors, *H. pylori* infection contributes to 90% of non-cardia gastric cancer, the most common subtype of gastric cancer [1]. Therefore, an improved understanding of the epidemiology behind the infection combined with healthier eating habits and smoking cessation have contributed to a steady decline in the prevalence of gastric cancer in the last 50 years. However, the five-year survival rate of gastric cancer is 31% because most cases have already metastasized at the time of diagnosis [1]. While screening methods are effective, low screening rates are a major contributor to late presentation and low survival with an increased risk of brain metastasis.

The presenting signs and symptoms of gastric cancer are variable, depending on the grade and stage of the cancer. The disease often begins with nausea, vomiting, epigastric pain, weight loss, and early satiety [5,7,12]. Brain metastasis can present with personality changes, cachexia, ataxia, and focal neurological deficits indicating late-stage gastric cancer [3,7]. Subsequent multidetector CT, endoscopic ultrasonography, and positron emission tomography are used to determine tumor depth, depth of wall invasion, and metastases [13]. Ultrasound may show a hypoechoic mass within the serosa of the stomach, whereas other modalities show annular masses causing gastric obstruction or adhering to the stomach [14]. Brain metastasis is observed as ring-enhancing lesions on MRI.

Some countries, such as Japan and South Korea, have a disproportionally higher incidence of gastric cancer due to the high prevalence of smoked foods in traditional cuisine [15]. These countries have implemented national gastric screening programs to combat the issue by providing contrast radiography screening and endoscopies. These programs have successfully decreased the incidence of gastric cancer in compliant patients, detecting up to 74% of cases of early gastric cancer [16]. Additionally, serum markers such as pepsinogen levels, *H. pylori* serology, and serum trefoil factor 3 have been used as diagnostic measures of gastric cancer [17]. A mental status exam (MSE) is warranted in each positive test to determine any neurological deviation that may suggest brain metastasis. The MSE indicates the cancer stage and prognosis of the patient.

After diagnosis, there are multiple treatment options depending on the extent of metastasis. Solitary brain metastases are treated with resection, radiotherapy, and chemotherapy [7]. However, late-stage gastric cancer, including metastases to the brain, is often treated with palliative care or surgical resection, as the prognosis at this point is poor [9]. Other common treatment options include SRS and WBRT [9].

Until now, treatment strategies involving an aggressive combination of these approaches have been lacking. A 40-year retrospective study at M.D. Anderson Cancer Center in Houston, Texas, reported 3,320 patients diagnosed with gastric cancer in that period with 0.7% metastasizing to the brain. The median survival time for these patients was only nine weeks due to few therapeutic options [9]. Another study demonstrated neurological improvement in 16.7% of patients treated only with surgical resection and/or WBRT, but no improvement in survival time compared to patients who received only pharmacologic monotherapy [7].

However, some studies report that alternative combinations of these therapies have increased the life expectancy of patients with brain metastases from nine weeks to one year [8,9]. After a thorough literature review, we believe our case is among the first to describe a patient who exhibited complete resolution of symptoms at a four-year follow-up. We hope these results encourage further research regarding the most effective treatment plan to improve the quality of life following the diagnosis of gastric cancer and prevent and manage metastasis to the brain.

## Conclusions

Gastric cancer with brain metastasis is a rare phenomenon associated with nonspecific neurological symptoms, including progressive focal neurological deficits, personality changes, and ataxia. It presents on MRI as variably enhancing lesions with internal hemorrhage and surrounding edema. Despite poor outlook following brain metastasis, we presented the case of a 73-year-old woman whose combination treatment of radiation therapy, capecitabine, decadron, and craniotomy eradicated the metastasis with no evidence of disease recurrence at the four-year follow-up. This represents a rare case of a patient successfully treated for gastric cancer with brain metastasis and mitigation of neurological symptoms. Therefore, we would like to prompt further research into the efficacy of pharmaceutical, surgical, and radiation combination therapy in patients with this condition.
